# Impact of cancer and chemotherapy on autonomic nervous system function and cardiovascular reactivity in young adults with cancer: a case-controlled feasibility study

**DOI:** 10.1186/s12885-015-1418-3

**Published:** 2015-05-18

**Authors:** Scott C. Adams, Ronald Schondorf, Julie Benoit, Robert D. Kilgour

**Affiliations:** 1 Department of Exercise Science, Concordia University, Montreal, QC Canada; 2 Department of Neurology, Jewish General Hospital, Montreal, QC Canada; 3 Behavioural Medicine Laboratory, Faculty of Physical Education & Recreation, University of Alberta, Edmonton, AB Canada

**Keywords:** Cancer, Autonomic nervous system, Composite autonomic scoring scale, Cardiovascular disease, Young adults

## Abstract

**Background:**

Preliminary evidence suggests cancer- and chemotherapy-related autonomic nervous system (ANS) dysfunction may contribute to the increased cardiovascular (CV) morbidity- and mortality-risks in cancer survivors. However, the reliability of these findings may have been jeopardized by inconsistent participant screening and assessment methods. Therefore, good laboratory practices must be established before the presence and nature of cancer-related autonomic dysfunction can be characterized. The purpose of this study was to assess the feasibility of conducting concurrent ANS and cardiovascular evaluations in young adult cancer patients, according to the following criteria: i) identifying methodological pitfalls and proposing good laboratory practice criteria for ANS testing in cancer, and ii) providing initial physiologic evidence of autonomic perturbations in cancer patients using the composite autonomic scoring scale (CASS).

**Methods:**

Thirteen patients (*mixed diagnoses*) were assessed immediately before and after 4 cycles of chemotherapy. Their results were compared to 12 sex- and age-matched controls. ANS function was assessed using standardized tests of resting CV (tilt-table, respiratory sinus arrhythmia and Valsalva maneuver) and sudomotor (quantitative sudomotor axon reflex test) reactivity. Cardiovascular reactivity during exercise was assessed using a modified Astrand-Ryhming cycle ergometer protocol. Our feasibility criteria addressed: i) recruitment potential, ii) retention rates, iii) pre-chemotherapy assessment potential, iv) test performance/tolerability, and v) identification and minimizing the influence of potentially confounding medication. T-tests and repeated measures ANOVAs were used to assess between- and within-group differences at baseline and follow-up.

**Results:**

The overall success rate in achieving our feasibility criteria was 98.4 %. According to the CASS, there was evidence of ANS impairment at baseline in 30.8 % of patients, which persisted in 18.2 % of patients at follow-up, compared to 0 % of controls at baseline or follow-up.

**Conclusions:**

Results from our feasibility assessment suggest that the investigation of ANS function in young adult cancer patients undergoing chemotherapy is possible. To the best of our knowledge, this is the first study to report CASS-based evidence of ANS impairment and sudomotor dysfunction in any cancer population. Moreover, we provide evidence of cancer- and chemotherapy-related parasympathetic dysfunction – as a possible contributor to the pathogenesis of CV disease in cancer survivors.

## Background

Despite therapeutic advances, cancer survivors remain at higher risk of disease- and treatment-related CV morbidity and mortality [1, 2]. Autonomic impairment, or neuropathy, is a nervous system disorder affecting the control of involuntary functions, including, digestion, heart rate, blood pressure, and perspiration. Preliminary associations between autonomic impairment-related CV dysfunction and increased risk/severity of CV disease and all-cause mortality have been proposed by a number of reviews of epidemiological- and clinical trial-based research [3–5]. Various anti-cancer chemotherapies may further affect the function of the autonomic and CV systems. Using both short duration and 24 h recordings, diminished heart rate (HR) variability has been reported in patients treated with vincristine [6], doxorubicin [7], various combination therapies [8, 9], and in some [10], but not all [11], patients treated with paclitaxel. Aberrant blood pressure variability and maladaptive orthostatic responses have been observed in patients treated with paclitaxel, taxanes, vinca alkaloids and cisplatin [12–15] – although the mechanisms were not always clear. However, these studies lacked consistency in their selection/execution of autonomic challenges, application of their eligibility and testing criteria. More specifically, several of these trials included participants with advanced age and pre-cancer comorbidities (i.e., diabetes and heart disease), both of which are known perturb ANS reflex responses. Furthermore, many failed to include key methodological details required to compare between trials. As such, they provide very little clinical relevance, and there remains insufficient evidence to make any conclusions regarding the presence or nature of cancer-related autonomic dysfunction.

Interestingly, regular aerobic exercise training has been shown to improve indices of CV health (i.e., HR variability) in various CV disease (CVD) populations [16–20]. As such, aerobic exercise may be effective in improving similar CV outcomes in cancer patients. Although a unifying mechanism of cancer-related CVD development has yet to be elucidated, a potential contributing factor may be the effects of cancer and anticancer therapies on ANS function. This relationship is often suggested in the literature but has yet to be clearly defined [3]. This line of investigation may be most important/relevant within the young adult cancer population for two reasons. First, ANS function is known to decline with age and is influenced by existing comorbidities [21]. By virtue of their age, young adult cancer patients are the most likely to have normal ANS reflexes. Second, given their average 5 years survival rates and greater number of years of life ahead of them [22, 23], the premature development of CVD in young adult cancer survivors is likely to account for many more years of life affected per individual.

The purpose of this study was to assess the feasibility of conducting concurrent ANS and CV evaluations in young adult cancer patients undergoing treatment for various cancers. However, we were concerned that, given the heterogeneity of the population (i.e., diagnoses and treatments) and the complexity of the reflex responses, ANS testing during cancer treatment may not provide reliable evidence of ANS dysfunction. Beyond this, our additional feasibility concerns included: i) recruitment potential (given that young adults account for only 10 % of cancer diagnoses, and we recruited at time of diagnosis), ii) retention rates (anticipated difficulty with compliance and follow-up), iii) capacity to establish a baseline assessment (variable time between initial diagnosis and commencement of systemic therapy), iv) performance and tolerability of the ANS and CV test battery components. Furthermore, we also sought to document and report the prevalence of confounding medication use, as they may perturb ANS and CV reflex responses in related lines of research. Our primary objectives were to identify the methodological pitfalls and propose good laboratory practice criteria for future autonomic testing in cancer. Our secondary – hypothesis generating – objective was to use modern clinical assessment techniques to provide evidence of autonomic perturbations in young adult cancer patients as a potential precursor to the development of CVD. According to pilot study guidelines [24], our research questions and methods were designed to reflect those to be used in a subsequent, larger investigation of the subject. Our primary research question was developed to determine if cancer or chemotherapy have a significant impact on ANS and CV function in young adult cancer patients. We hypothesized that young adults with cancer would demonstrate an increased incidence and severity of cancer- and chemotherapy-related ANS and CV dysfunction (vs. controls); and, that the ANS dysfunction would significantly impair the exercise response of young adult cancer patients to, and in recovery from, a brief submaximal exercise challenge.

## Methods

Recruitment took place from March 2010 to July 2011. Eligible patients with all stages of disease, aged 18–45 with an Eastern Cooperative Oncology Group performance status (ECOG) ≤ 2, were recruited from the McGill Adolescent and Young Adult Oncology Program and the Segal Cancer Centre of the Jewish General Hospital, Montreal Quebec. Healthy control subjects (*age- and gender-matched hospital staff and university students*) were recruited by word of mouth, on a case-by-case basis. *Exclusion criteria*: i) use of any medications, at T1, that interfered with autonomic or CV function, ii) intrinsic cardiac disease or ANS-perturbing comorbidity (e.g., arrhythmia, intraventricular conduction defects, evidence of cardiac ischemia, pre-existing cardiomyopathy, diabetes, hypertension, neuropathy, seizure disorder) and iii) an inability to perform any of the baseline (T1) ANS or CV challenges due to tumor location. In accordance with our feasibility objectives, detailed records of recruitment, retention, testing and confounding medication use were kept. Jewish General Hospital and Concordia University institutional review boards both approved this study (*protocol # 04–032*).

Oncologist clearance and verbal patient consent were obtained prior to explaining the study. Patients reviewed the informed consent and were able to ask questions. Those agreeing were given detailed pre-test instructions according to best practices of ANS and CV testing [21, 25]. T1 was booked within 24 h of recruitment. Informed consent was signed at T1. All patients underwent ANS and CV evaluations at T1 (*post-diagnosis and pre-chemotherapy*) and follow-up (T2; *after their 4th, and one week prior to their 5th chemotherapy treatment – hypothesized to be the intra-treatment period least susceptible to the influence of confounding medication use*).

**Fig. 1:**
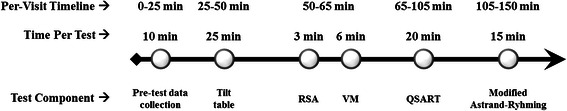
Sequence of tests

All procedures were conducted within the hospital’s Autonomic Reflex Laboratory. Self-reported fatigue was measured using the Brief Fatigue Inventory [26] – comprised of 10 questions, scored from 0–10, with a total possible score of 100 arbitrary units (a.u.). Self-reported physical activity levels were reported and expressed as MET∙hrs∙week^−1^. Following standard protocols [21], the non-invasive battery of tests used at rest (respiratory sinus arrhythmia (RSA), Valsalva maneuver (VM), tilt-table and quantitative sudomotor axon reflex test (QSART)) provided information concerning cardiovagal, sympathetic adrenergic vasomotor and cardiomotor, as well as postganglionic sympathetic cholinergic sudomotor function [27, 28]. The severity and localization of the type and sites of autonomic dysfunction were graded and compared using a validated composite autonomic scoring scale (CASS) [27, 28]. Immediately following the resting ANS protocol, the subjects were transferred to an adjacent evaluation room to perform a brief, 6-min, submaximal exercise challenge on a cycle ergometer [29] (Fig. 1). The exercise test was proposed to obtain a functional assessment of the indices of CV function (e.g., central (e.g., HR variability, HR, stroke volume and cardiac output) and peripheral (blood pressure and systemic vascular resistance)) that correlate with the ANS testing.

In establishing our feasibility criteria (FC), several important factors were weighed. First, previous investigations of ANS function in cancer [6, 8–10, 12, 13, 30–33] often had small sample sizes, did not establish pre-treatment baselines, included a wide age-range of participants and lacked sufficient and consistent methodological and results reporting. Second, in accordance with pilot study guidelines [24], and given that our trial did not include an intervention, we established more stringent FC to reflect the factors that could hinder a larger, more definitive trial. Therefore, in the absence of good laboratory practices for ANS testing in cancer, we based most decisions for our FC on the combined results of recent, in-treatment, randomized controlled cancer-exercise trials [34–37] and McGill Adolescent and Young Adult Oncology Program clinic data [Palumbo M, Kavan P: Adolescent and Young Adult Oncology: The Challenge in Serving a Unique, Underserved Population - Five Year Experience of The McGill University Adolescent and Young Adult Oncology Program, Unpublished 2008 – 2013] which included comprehensive reports of key methods, recruitment strategies, trial design and participant flow. In the end, our feasibility criteria addressed i) patient recruitment and access (i.e., (a) prevalence of baseline comorbidities and confounding medication use, and (b) identification of eligible patients), ii) subject retention rates, iii) capacity to establish a post-diagnosis/pre-chemotherapy baseline (i.e., (a) number of days between diagnosis and initiation of treatment, and (b) any time-related testing constraints (e.g., scheduling conflicts, physician availability)), iv) test performance, v) test tolerability, and vi) prevalence of potentially confounding medication use.

Severity of autonomic dysfunction was assessed using the CASS [27]. Spectral analysis of HR and baroreflex function assessed at rest and during head-up tilt, using established methods and procedures [38–40], provided additional indices of the integrity of cardiac autonomic and sympathetic vasomotor innervation [40, 41]. To evaluate the influence of disease proliferation on our endpoints, baseline comparisons were made using standard t-tests. To assess the impact of exposure to chemotherapy on our endpoints, group-by-time interactions were evaluated using 2x2 repeated measures ANOVAs. Based on recent pilot study methodological recommendations, the feasibility-nature of the investigation, and the anticipated small size and heterogeneous nature of our sample, a power analysis, subgroup analyses or adjustments for relevant covariates and potential effect modifiers were not performed [24]. Furthermore, given the feasibility-nature of the study, significance was set at α = 0.05 and not adjusted for multiple comparisons.

## Results

### Primary results: feasibility outcomes

We were 98.4 % successful in achieving our target FC (Table 1).

**Table 1:** Feasibility results

Feasibility Criteria	Target	Actual	% Attained
I. Patient recruitment & access			
I.i Comorbidity & medication free	< 5 %	3.6 % (1/28 OEP)	100 %
I.ii Patient identification rate	>40 patient/year	>46 patients/year	100 %
II. Subject retention	>95 %	92.3 %	97.2 %
III. Baseline establishment			
III.i Days between diagnosis & T1	≤9 days	9.1 days	99.9 %
III.ii Time-related testing constraints	<20 % missed	27 % missed	91 %
IV. Test performance & tolerability			
IV.i Test performance	>95 % test completion	94.3 test completion	99.3 %
IV.ii Testing tolerance	<1 % AEs/< 10 % ACs	0.83 % AE / 5.1 % AC	100 % / 100 %
V. Potentially confounding medication use	N/A	T1: 15.4 % T2: 83.3 %	

*OEP* otherwise eligible participants, *AE* adverse events, *AC* active complaints

### Secondary results: exploratory self-report and physiology outcomes

#### Baseline demographic and self-report results

**Fig. 2:**
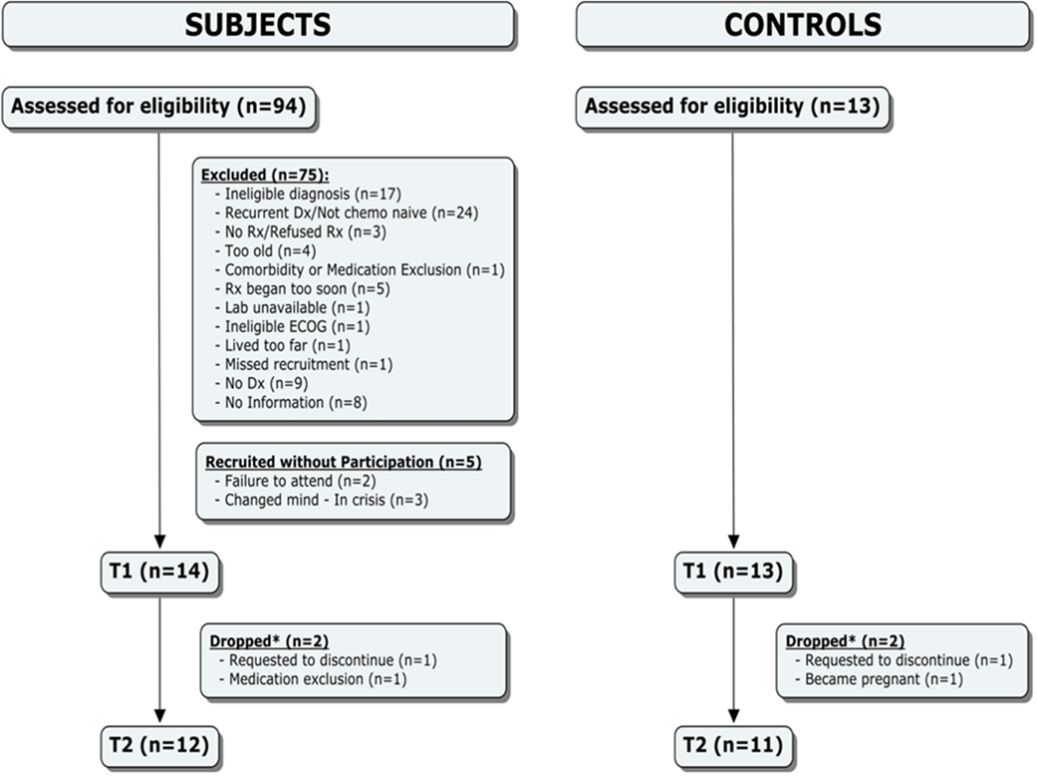
Subject recruitment and testing

Thirteen cancer patients and 12 sex-and age-matched controls were tested at T1 (Fig. 2). Subject demographic and medical information is presented in Tables 2, 3 and 4. Study groups were closely matched in gender, age and body mass index. There was a significant difference at T1, t(21) = 2.594, p = 0.017, in weekly physical activity levels (mean ± SD) between the patient (7.5 ± 7.6 MET∙hrs∙week^−1^) and control groups (26.3 ± 22.9 MET∙hrs∙week^−1^), respectively. There was also a trend towards significance in T1, t(21) = 2.066, p = 0.051, in Brief Fatigue Inventory scores (*mean ± SD;* out of a possible 100 a.u.) between the patient (27.3 ± 14.2 a.u.) and control groups (15.5 ± 13.2 a.u.), respectively.

**Table 2:** Baseline subject characteristics

	Control mean ± SD	Patient mean ± SD
n	11	12
Sex (women)	55 % (n = 6)	67 % (n = 8)
Age (years)	33.8 ± 8.1	35.0 ± 8.9
Weight (kg)	66.3 ± 12.5	66.5 ± 11.5
BMI (kg/m^−2^)	23.0 ± 2.1	23.0 ± 3.5
BFI	15.5 ± 13.2	27.3 ± 14.2^b^
PA level (*MET · hrs∙week*^*−1*^*)*	26.3 ± 22.9	7.5 ± 7.6^a^

*SD*standard deviation, *BMI* body mass index, *BFI* brief fatigue inventory, *PA*physical activity, *MET* metabolic equivalent of task

^a^p ≤ 0.05; ^b^p = 0.051

**Table 3:** Patient diagnosis and treatment characteristics

Cancer types	Treatment protocol (*# of patients*)
Breast	4 cycles FEC + Paclitaxel (n = 1)
	4 cycles AC (n = 4)
Gastrointestinal	
Pancreatic	FOLFIRINOX x 3 + Gemcitabine x 1 *(n = 1)*
Colon	Xelox x 4 *(n = 1)*
Anal	Mitomycin C + 5 FU x 4 *(n = 1)*
Hematological	
Hodgkin’s Lymphoma	ABVD *(n = 2)*
Non-Hodgkin’s Lymphoma	R-CHOP *(n = 1)*
Other	
Adenocarcinoma (unknown origin)	C-Pacli *(n = 1)*
Parotid (acinic cell carcinoma)	C-Pacli *(n = 1)*

*FEC* fluorouracil epirubicin cyclophosphamide, *AC* adriamycin cyclophosphamide, *FOLFIRINOX*, folinic acid fluorouracil irinotecan oxaliplatin, *5 FU* fluorouracil, *ABVD* adriamycin bleomycin vinblastine dacarbazine, *R-CHOP* rituximab + cyclophosphamide hydroxyldaunoreubicin oncovin (vincristine) prednisone, *C-Pacli* carboplatin + paclitaxel

**Table 4:** Patient disease staging and functional status

		# of Patients (%)
Staging at diagnosis	Stage I	1 (8)
	Stage II	6 (46)
	Stage III	3 (23)
	Stage IV	3 (23)
Performance status (0–4)	Baseline	
	ECOG 0	3 (23)
	ECOG 1	8 (62)
	ECOG 2	2 (15)
	Follow-up	
	ECOG 0	4 (33)
	ECOG 1	7 (58)
	ECOG 2	1 (8)

#### CASS results

**Fig. 3:**
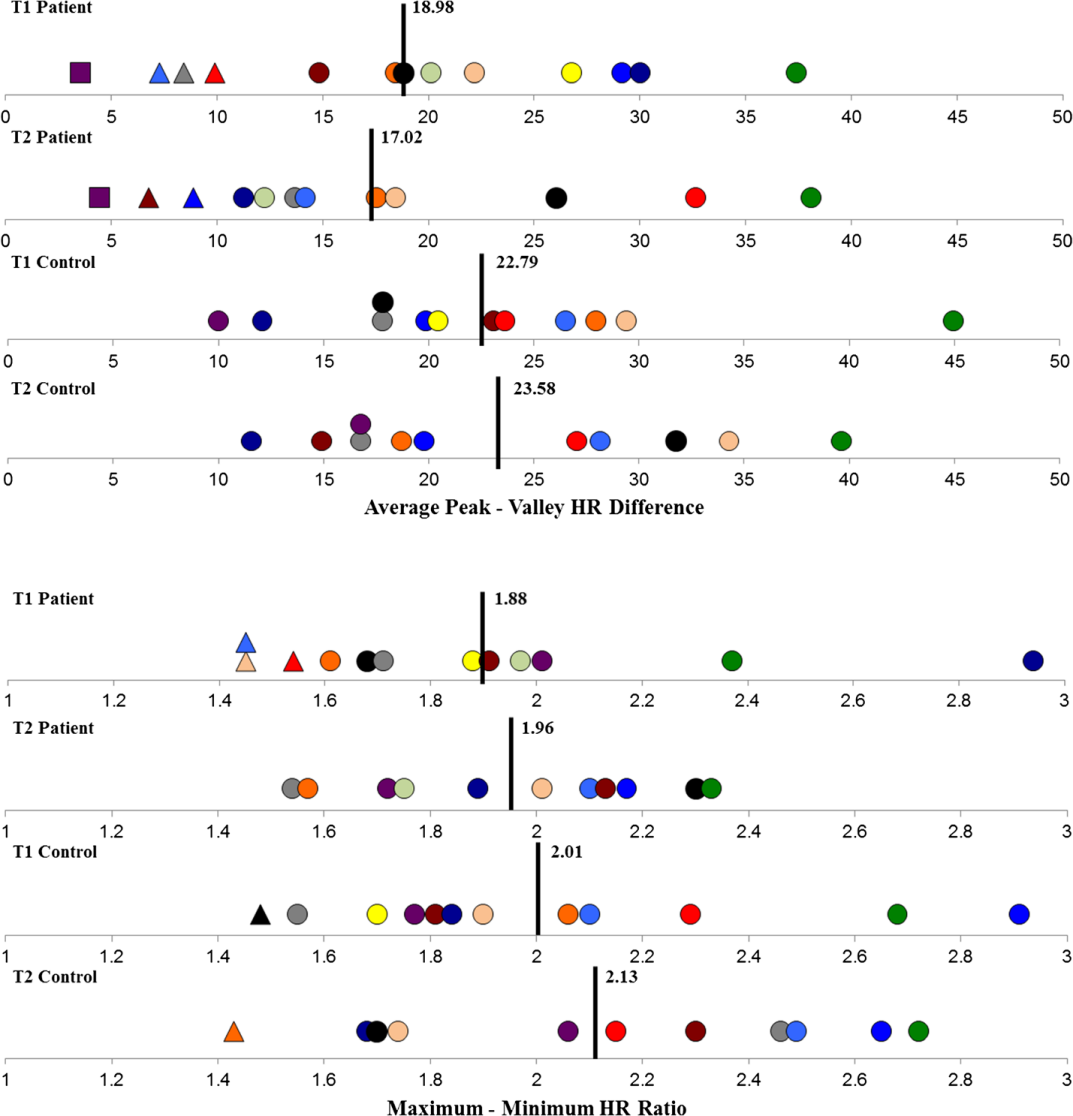
Individual RSA (*top*) and Valsalva Ratio (*bottom*) scores for patients and controls at baseline and follow-up. *Note* Data points have been color-coded within each group and according to subject. Circular data points reflect scores or measurements falling within normal age- and gender-related ranges. Whereas triangular and square data points reflect scores or measurements > 50 % and < 50 %, respectively, of the lower normal age- and gender-related limits. Black vertical bars and corresponding values represent group means for each time point

**Fig. 4:**
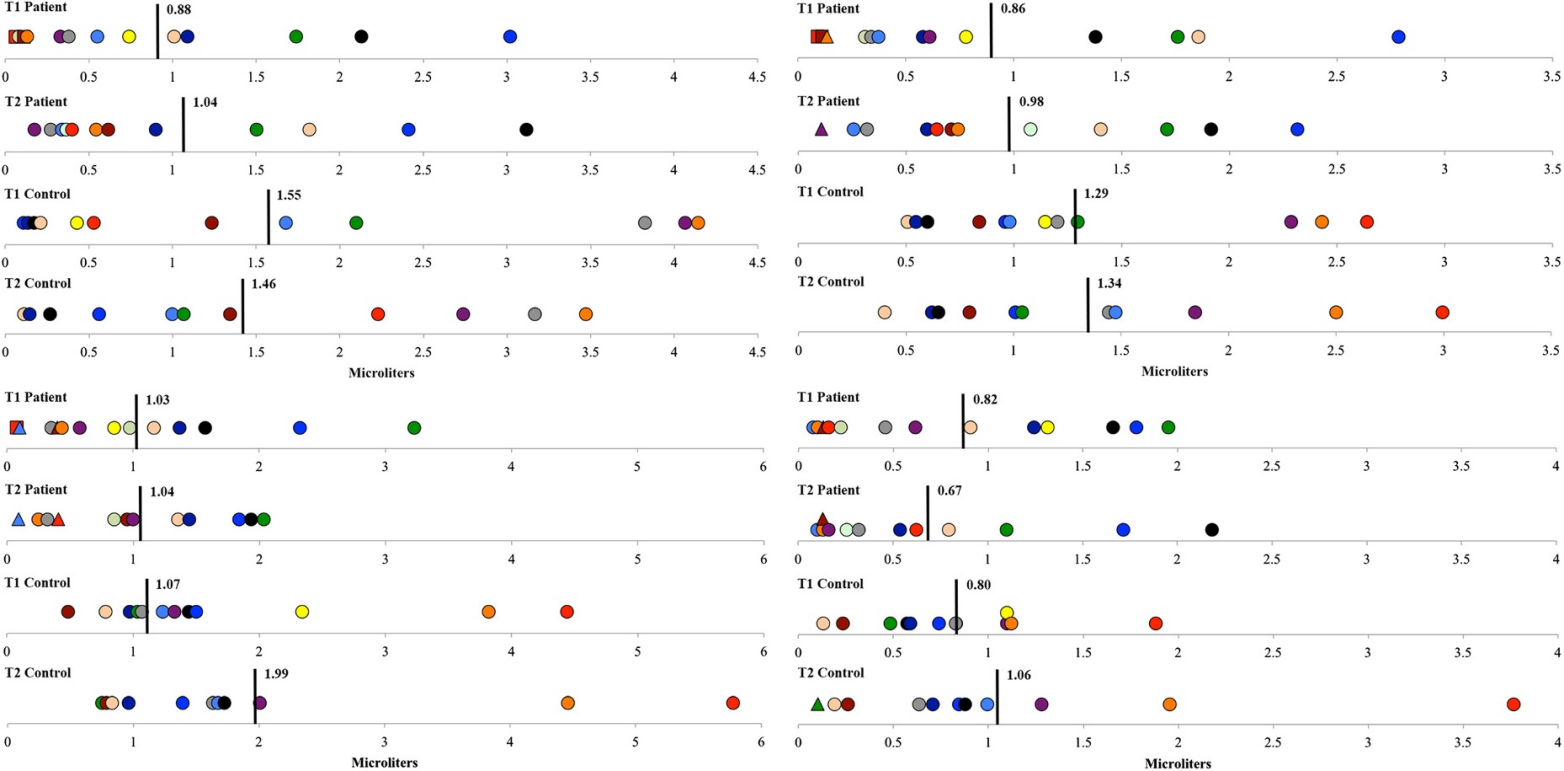
Individual QSART scores at the forearm (*top left*), proximal leg (*top right*), distal leg (*bottom left*) and foot (*bottom right*) for patients and controls at baseline and follow-up. *Note* Data points have been color-coded within each group and according to subject. Circular data points reflect scores or measurements falling within normal age- and gender-related ranges. Whereas triangular and square data points reflect scores or measurements > 50 % and < 50 %, respectively, of the lower normal age- and gender-related limits. Black vertical bars and corresponding values represent group means for each time point

T2 measurements were collected a mean of 14.3 weeks and 19.0 weeks after T1 for patients and controls, respectively. 2x2 repeated measures ANOVAs revealed significant, or near significant, between-group differences for all dependent variables found in Table 5. Autonomic dysfunction is defined as a minimum score of two in any of the three CASS domains (i.e., cardiovagal, adrenergic and sudomotor), or a minimum score of one in at least two domains – out of a total possible score of 10 [42]. After applying the CASS criteria, the individual (Figs. 3 and 4) and group results (Tables 6 and 7) demonstrate mild to moderate ANS dysfunction at T1 (1.23 ± 1.59; *mean ± SD*), with slight improvement in ANS function at T2 (0.67 ± 0.99). The observed group main effects were the patients’ cardiovagal [F(1,21) = 4.575, p = 0.044] and total [F(1,21) = 5.975, p = 0.023] CASS scores were significantly higher than controls. Although the difference between patients’ and controls’ sudomotor CASS scores did not reach significance [F(1,21) = 3.702, p = 0.068], the number of patients who had abnormal or borderline abnormal QSART scores was significantly higher than controls [F(1,21) = 4.830, p = 0.039]. Neither group displayed evidence of severe ANS or CV dysfunction at either testing time point. Unmanaged, disease- and treatment-related complications prevented one patient from attempting the VM at T1, and a different patient from attempting the VM and tilt-table test at T2. Two patients and two controls demonstrated orthostatic intolerance during the tilt-table test at T1 and T2 (*one subject from each group at each time point*).

**Table 5:** Main group effects

Test component	Control mean ± SE	Patient mean ± SE	Mean difference (95 % CI)	p	Partial eta^2^
Tilt-Table HR Differences					
Baseline	56.39 ± 3.02	73.07 ± 2.88	16.7 (8.0 to 25.4)	0.001	0.46
Tilt 1	75.9 ± 4.09	93.48 ± 3.90	17.6 (5.8 to 29.4)	0.006	0.34
Tilt 2	78.6 ± 4.09	95.2 ± 3.90	16.6 (4.8 to 28.4)	0.008	0.31
Tilt Total	77.26 ± 4.05	94.34 ± 3.87	17.1 (5.4 to 28.8)	0.007	0.33
Bike HR Differences					
Baseline	69.85 ± 3.92	81.27 ± 3.92	11.4 (−0.2 to 23.1)	0.054	0.19
Warm-up	73.36 ± 4.42	84.53 ± 4.42	11.2 (−1.9 to 24.3)	0.09	0.15
Loading	78.99 ± 4.27	88.59 ± 4.27	9.6 (−3.1 to 22.3)	0.13	0.12
2nd minute	121.5 ± 5.75	128.53 ± 5.75	7.0 (−10.1 to 24.1)	0.399	0.04
6th minute	137.1 ± 4.83	144.8 ± 4.83	7.7 (−6.6 to 22.1)	0.274	0.07
Stop	109.36 ± 4.79	124.71 ± 4.79	15.3 (1.1 to 29.6)	0.036	0.22
Cool down	91.9 ± 4.83	106.1 ± 4.83	14.2 (−0.2 to 28.5)	0.052	0.19
QSART					
*Baseline Sweat Rates*					
Forearm	70.96 ± 5.03	62.13 ± 4.82	−8.8 (−23.3 to 5.7)	0.219	0.07
Proximal Leg	66.68 ± 4.86	57.75 ± 4.65	−8.9 (−22.9 to 5.1)	0.198	0.08
Distal Leg	68.23 ± 4.97	59.42 ± 4.76	−8.8 (−23.1 to 5.5)	0.214	0.07
Foot	83.68 ± 4.86	68.17 ± 4.65	−15.5 (−29.5 to −1.5)	0.031	0.20
# Sites <10 % of normal	0.18 ± 0.31	1.13 ± 0.30	0.95 (0.05 to 1.8)	0.039	0.19

*SE*,standard error; *CI*, confidence interval

**Table 6:** CASS Component Scores

	T1					T2				
	# Tested n	# Mild n (%)	# Moderate n (%)	# Severe n (%)	% Affected	# Tested n	# Mild n (%)	# Moderate n (%)	# Severe n (%)	% Affected
Sudomotor function										
Patients	13	2 (15.4)	0 (0.0)	2 (15.4)	30.8	12	4 (33.3)	0 (0.0)	0 (0.0)	33.3
Controls	12	0 (0.0)	0 (0.0)	0 (0.0)	0	11	1 (9.1)	0 (0.0)	0 (0.0)	9.1
Cardiovagal function										
Patients	12	0 (0.0)	2 (16.7)	3 (25.0)	41.7	11	2 (18.2)	1 (9.1)	0 (0.0)	27.3
Controls	12	1 (8.3)	0 (0.0)	0 (0.0)	8.3	11	1 (9.1)	1 (9.1)	0 (0.0)	18.2
Adrenergic function										
Patients	11	0 (0.0)	0 (0.0)	0 (0.0)	0.0	12	0 (0.0)	0 (0.0)	0 (0.0)	0.0
Controls	12	0 (0.0)	0 (0.0)	0 (0.0)	0.0	11	0 (0.0)	0 (0.0)	0 (0.0)	0.0

Normative values taken from [66, 67]

**Table 7:** CASS scores

CASS	T1				T2			
	Tested	Mild Neuropathy n (2–4)	Moderate Neuropathy n (5–7)	% Affected	Tested	Mild Neuropathy n (2–4)	Moderate Neuropathy n (5–7)	% Affected
Patient	13	3	1	30.8	12	2	0	18.2
Control	12	0	0	0.0	11	0	0	0.0

#### Exercise testing results

At T1, predicted maximal oxygen uptake (VO_2max_) was significantly lower in the patient group (2.54 ± 0.70 ml O_2_∙min^−1^; *mean ± SD*) than in controls (3.37 ± 0.99 ml O_2_∙min^−1^, t(19) = 2.172, p = 0.043). The observed group main effects was the patients’ predicted VO_2max_ (2.52 ± 0.36 ml O_2_∙min^−1^; *mean ± SE*) was significantly lower than controls (3.36 ± 0.36 ml O_2_∙min^−1^; F(1,18) = 5.493, p = 0.031). In addition, compared to controls, patients’ HR recovery improved significantly following treatment [F(1,15) = 4.938, p = 0.042], which mirrored the direction of change in the CASS score. Again, disease- and treatment-related complications prevented one patient from attempting the exercise test at T1, and a different patient from attempting the exercise test at T2.

## Discussion

In the present study, we aimed to establish recommendations and guidelines upon which future investigations of ANS function in cancer could be planned and implemented. The principle finding of a combined 98.4 % success in achieving our FC suggests that a larger scale investigation of cancer-related autonomic dysfunction in young adults is possible.

Acute and chronic autonomic impairments have deleterious effects on quality of life and survival in health and a variety of disease states [3]. Initial evidence of cancer- and chemotherapy-related ANS dysfunction have been shown here and in the literature (both during and post-treatment) [6–10, 12, 30, 32, 33, 43–48]. However, the methodology in previous ANS-cancer research has been inconsistent. Therefore, we based our feasibility assessment upon methodologically robust exercise oncology trials. This basis may be particularly relevant given the potential for using exercise as a modality to preserve and restore ANS and CV function in cancer, as has been described in other populations [49]. In an attempt to inform good laboratory practice, and drawing from our collective experience, we propose the following methodological considerations to facilitate future studies of autonomic function in cancer (Table 8).

**Table 8:** Good laboratory practice recommendations and considerations for ANS and CV testing in cancer

Patient recruitment, access and retention	1. Recruit young adults from expanded age range (*18*–*45* vs. *18–39*).
	• *Boosts recruitment opportunity without exposing sample to preexisting ANS/CV morbidity and use of related medications.*
	*2.* Prescreen for age and diagnosis (*given the absence of underlying confounding morbidity*).
	3. YA cancer patients appear highly motivated and capable of participating in studies of this nature.
	• Observational results - therefore, cannot generalize findings to intervention trials.
Pre-Chemotherapy baseline	4. Ensure unrestricted testing facility access.
	• YAs are at a higher risk of late- and misdiagnosis [68, 69], which may increase the likelihood of advanced staging at diagnosis and shorten the available pre-treatment testing window.
	• Investigators should make every attempt to anticipate, screen for and test patients prior to their beginning pre-treatment symptom management medication.
Test rerformance and tolerability	5. Proceed with CASS test battery as described.
	• No differences in test tolerability or performance between patients and controls.
Use of potentially confounding medication	6. Identify an assessment window which minimizes the influence of PCM.
	• For 2–4 weeks/cycle treatment protocols, assessments should be made between 3 and 6 days prior to subsequent treatment cycle.
	• For 1 week/cycle treatment protocols, coordinating with the treating oncologist may be required to establish the requisite assessment opportunity.
	7. Record and report PCM use in all observation and intervention trials. • In addition to primary anti-cancer treatments and commonly prescribed symptom-management medications (i.e. anti-emetic and pain-management), we identified the occasional use of additional medications (i.e. antidepressant and sleep-aids) which may influence ANS and CV testing.
Additional considerations	8. Systemic anticancer therapies are known to injure/perturb multiple CV system components.
	∴Aberrant ANS test results may reflect end-organ dysfunction and not ANS dysfunction.
	∴Include complementary CV and end-organ function tests in ANS-oncology research.
	9. The single stage Astrand-Ryhming may lack sensitivity.
	∴ Consider a submaximal or maximal incremental ramp exercise protocol instead.
	10. Investigators should assess and account for differences in aerobic fitness, physical activity and fatigue levels, given their relationships with measures of ANS function [3, 47, 51, 53].

In keeping with our stated objective to facilitate hypothesis generation for future research, the findings of this exploratory investigation include significant between-group differences in cardiovagal and total CASS scores and a significantly increased prevalence of sudomotor dysfunction – potentially suggestive of mild to moderate, cholinergic-mediated, impairment of ANS function in our patient group versus controls. We also report, compared to controls, significantly greater HRs during the tilt table (T1-T2) and Astrand-Ryhming (*baseline and recovery periods*) challenges (T1-T2), and lower predicted VO_2max_ (T2 only). We observed a slight improvement in ANS and CV function at T2 (*CASS and post exercise HR recovery*). However, given the cumulative nature of chemo-toxicity, it is possible that greater treatment effects would have been observed if T2 measures were collected following treatment vs. just four cycles.

The cardiovagal, adrenergic, and sudomotor CASS components reflect the integrity of parasympathetic, sympathetic, and sudomotor sympathetic branches of the ANS, respectively [27]. Supporting the evidence for paraneoplastic-related CV and autonomic dysfunction in cancer [9, 30, 33, 47, 50, 51], our baseline assessment revealed evidence of CV and ANS differences between groups. Higher patient HRs obtained throughout the tilt table test, as well as at baseline and in recovery from the bike test, may reflect inadequate parasympathetic (or acetylcholine-mediated) restraint of HR. Alternatively, the observed HRs differences may be an artifact of the poorer aerobic fitness levels of our patient group and not be autonomically-mediated. In addition, there was a significant between-group difference in the number of abnormal QSART sites in our patient group (vs controls). This too may reflect an acetylcholine-mediated mechanism of ANS dysfunction. Contrary to reports of increased ANS impairment during or following treatment [30, 33], and similar to the findings of Turner et al. [9], our results demonstrated persistent, yet slightly improved, ANS dysfunction in our patient group at T2. Given the evidence supporting the correlation between higher resting parasympathetic tone and higher aerobic fitness levels [51–58], the potentially confounding influence of aerobic fitness levels between groups must be considered when interpreting our cardiovagal results. Contrary to reports of chemotherapy-induced attenuation of HR variability [6, 32, 43], spectral analysis of short-term HR variability recordings in our study (obtained during pre-tilt rest) did not reveal any observable fluctuation in parasympathetic activity (as indicated by the high-frequency domain). This is consistent with the findings of Ekholm et al. [10], who suggested that the acute chemotherapy-induced changes may be more subtle and therefore not as easily detected using short-term (vs. 24 h) recordings. Finally, to the best of our knowledge, we are the first to report QSART evidence of sudomotor impairment in cancer. Although there was a slight improvement in sudomotor function at T2 (shift in CASS scores toward more mild impairments), our assessment revealed a slight shift in which five out of the eleven patients were affected.

The effect of ANS impairments on survivorship and long-term quality of life remain unclear. Related research has demonstrated persistent ANS impairment in cancer survivors, with and without advanced disease [8, 32, 43, 46, 47]. However, the reported methods varied in their inclusion/exclusion criteria, ANS assessment techniques, and failed to control for known confounding comorbidities (i.e., diabetes and CVD) and use of related medications (i.e., various cardiac, antihypertensive and opioids). Furthermore, in the absence of a pre-treatment baseline evaluation and the inclusion of a wide age-range of participants (19–79 years), it is difficult to assess whether the reported ANS impairments have resulted directly from the various cancers or anti-cancer therapies. If, in fact, cancer(s) and anticancer therapies are responsible for causing long term ANS impairment or predisposing cancer survivors to related morbidity, the development of protective therapies is imperative.

CV exercise training has been shown to preserve and improve markers of ANS and CV health in other populations [49]. However, dysfunction of either the parasympathetic, sympathetic or sudomotor ANS branches may independently compromise the ability of affected cancer patients to exercise. More specifically, the shift toward a “sympathetic dominant ANS balance”, caused by either vagal withdrawal or sympathetic hyperactivity, predisposes individuals to chronically higher resting metabolic rates [3, 49], and therein an energetically unfavorable state. Furthermore, parasympathetic damage may hinder early exercise adaptations to [59, 60] and recovery rates from [61, 62], exercise. Inadequate sympathetic adjustments are known to cause maladaptive blood pressure responses [21] and may limit the attainment of maximal exercise performance [60, 63, 64]. Finally, aberrant sweat responses, resulting from sudomotor dysfunction, may compromise thermoregulation and place exercising cancer patients at risk for heat injury [65] and related exercise intolerance. Together, this evidence suggests that ANS dysfunction may variably affect the exercise capacity of patients attempting to engage in it. It is also important to consider that, even if exercise capacity is unaffected, ANS dysfunction could potentially be an effect modifier for exercise and multiple health outcomes. Drawing from this discussion, future research should explore and account for both the direct- and moderator-effects of cancer-related ANS dysfunction on established exercise-health outcome relationships. Unfortunately, due to limitations in our study design, we were unable to provide evidence of any relationship between CV performance changes and the observed ANS dysfunction. Possible reasons for this include: i) insensitivity of the modified, single stage Astrand-Ryhming protocol, ii) small sample size, and iii) wide range of normal biologic variability within our control group.

Given the potential confounders of ANS testing in cancer, and in an attempt to help localize cancer- and treatment-related damage, it is likely necessary to conduct complementary assessments effector organ function (i.e. pulmonary, cardiac and vascular function tests). Importantly, future investigations of autonomic and CV function should make every effort to minimize the influence of/report PCM use. Furthermore, to standardize the evaluation of ANS function in cancer, we strongly recommend the use of the CASS and components therein. Finally, contrary to our hypotheses, exposure to chemotherapy did not appear to cause significant, additional impairment of the ANS and CV responses to the CASS and exercise challenges. Although not statistically supported, our main findings (diminished RSA and QSART) may suggest a common cholinergic mechanism of dysfunction. When considering the proposed mechanism of acetylcholine and vagal function inhibition of proinflammatory cytokine release [3], it is interesting to speculate that if cancer-related parasympathetic dysfunction does exist, it may provide a pathway for CVD development in cancer patients.

### Limitations

As stated in the introduction and methods, the primary purpose of this study was to assess the feasibility of conducting concurrent ANS and CV assessments in young adults undergoing treatments for various cancers. In keeping with pilot study recommendations [24], we did not attempt to control for numerous confounders and covariates of ANS function when presenting our hypothesis-generating, physiology findings. Additional study limitations include: i) heterogeneity of diseases and related-treatments, ii) reliance on predicted, and not measured, VO_2max_ scores as indicators of aerobic fitness, iii) inaccurate patient reported outcomes, iv) the use of the Brief Fatigue Inventory to compare across cancer and non-cancer populations, and v) unreported pre-test protocol violations and use of PCMs. Therefore, we encourage readers to interpret all of the reported physiology findings with extreme caution.

## Conclusions

The general aim of this study was to establish the feasibility of testing autonomic and CV function in young adult cancer patients undergoing treatment for cancer by: i) identifying the methodological pitfalls and proposing good laboratory practice criteria for ANS testing in cancer, and ii) providing exploratory evidence of autonomic perturbations in cancer patients using the CASS. From a logistical standpoint, with a 98.4 % average success rate in achieving the targeted FC, we are confident that future investigations of autonomic function in cancer are possible. Cancer and chemotherapy are known to impact effector organ and nervous system function. However, due to the multiplicity of confounders on ANS testing (i.e., lifestyle, age, comorbidity, cancer type, and use of various anticancer therapies/supportive medications), the study of ANS function in cancer remains challenging. As such, rigorous research, involving the simultaneous evaluation of multiple CV system components (i.e., effector organs, vasculature and nervous system), is required to identify the mechanisms underlying the observed acute and long-term CVD morbidity and mortality risks in cancer survivors.

## Abbreviations

AC: Active complaints
AE: Adverse events
ANS: Autonomic nervous system
CASS: Composite autonomic scoring scale
CV: Cardiovascular
CVD: Cardiovascular disease
ECOG: Eastern cooperative oncology group performance status
FC: Feasibility criteria
HR: Heart rate
Kg: Kilograms
MET: Metabolic equivalent of task
Min: Minute
O_2_: Oxygen
OEP: Otherwise eligible patients
PCM: Potentially confounding medication
Pt: Patients
QSART: Quantitative sudomotor axon reflex test
RSA: Respiratory sinus arrhythmia
T1: Baseline testing
T2: Follow-up testing (post 4 cycles of chemotherapy)
VM: Valsalva maneuver
VO_2max_: Maximal oxygen uptake
